# The role of C-reactive protein in predicting all-cause mortality among Chinese arthritis patients: implications for public health education and promotion

**DOI:** 10.3389/fpubh.2025.1511860

**Published:** 2025-02-06

**Authors:** Shuchao Ye, Damei Ye, Changyi Lin, Dongming Lu, Xuelan You, Chaoyan Xu, Yongyang Wu

**Affiliations:** ^1^Department of Urology, Affiliated Sanming First Hospital, Fujian Medical University, Sanming, China; ^2^Department of Rheumatology Immunology, Affiliated Sanming First Hospital, Fujian Medical University, Sanming, China

**Keywords:** arthritis, C-reactive protein, all-cause mortality, China health and retirement longitudinal study, public health education

## Abstract

**Objective:**

Arthritis poses a significant public health challenge, contributing to increased healthcare resource utilization and reduced quality of life. C-reactive protein (CRP), a key inflammatory biomarker, plays a critical role in monitoring disease progression and guiding health promotion strategies. This study aims to investigate the association between CRP levels and all-cause mortality in Chinese arthritis patients, highlighting its implications for public health education and intervention programs.

**Methods:**

A prospective cohort study was conducted using data from the China Health and Retirement Longitudinal Study (CHARLS) from 2011 to 2020. Individuals diagnosed with arthritis in 2011 were included, with CRP levels as the primary exposure variable and mortality as the outcome of interest. Kaplan–Meier survival curves and Cox proportional hazards regression models were employed to assess the relationship between CRP levels and mortality risk, emphasizing the potential for targeted health education and promotion interventions.

**Results:**

A total of 3,413 participants were analyzed, with 87 deaths recorded during the 10-year follow-up period. Deceased individuals were older and exhibited higher levels of CRP, creatinine, and uric acid, alongside lower BMI, MET, eGFR, and HGB. Across three Cox regression models, elevated CRP levels (≥3 mg/L) were significantly associated with increased mortality risk [hazard ratio (HR) = 3.73 (2.23–6.23), HR = 3.00 (1.79–5.01), HR = 4.94 (1.77–13.78), respectively]. Kaplan–Meier survival curves further confirmed that arthritis patients with CRP levels ≥3 mg/L faced a markedly higher mortality risk.

**Conclusion:**

Elevated CRP levels are strongly associated with increased all-cause mortality in arthritis patients, underscoring the importance of integrating CRP monitoring into public health education and promotion strategies. Efforts to control inflammation and promote health literacy regarding arthritis management may improve survival outcomes and reduce the public health burden associated with arthritis.

## 1 Introduction

With an aging population and increasing life expectancy, arthritis has become one of the most burdensome musculoskeletal diseases, posing significant challenges to public health systems worldwide (1–3). Osteoarthritis (OA) and rheumatoid arthritis (RA) are the most prevalent forms of arthritis, with OA affecting 10% of men and 19% of women over the age of 60 (4). In China, rheumatoid arthritis has a prevalence of 0.5% among adults, ranking it among the top ten chronic diseases (5). These conditions progressively damage joints and synovial tissues, leading to symptoms such as pain, stiffness, swelling, and even deformity and functional loss (6). Beyond their direct impact on physical health, arthritis is associated with an increased risk of cardiovascular diseases and other complications, contributing to higher all-cause mortality rates (7). Identifying arthritis patients at high risk of mortality is therefore critical for developing targeted public health interventions and improving health outcomes.

C-reactive protein (CRP), an acute-phase reactant protein primarily produced by hepatocytes, is a well-established marker of systemic inflammation (8, 9). Elevated CRP levels have been linked to various adverse health outcomes, including cardiovascular disease and mortality. A systematic review of 23 cohort studies identified CRP, along with other biomarkers such as BNP and WBC, as significant predictors of all-cause and cause-specific mortality, including cardiovascular and cancer-related deaths (10). In arthritis patients, chronic inflammation leads to persistently elevated CRP levels compared to non-arthritis individuals, suggesting its potential role as a predictor of mortality risk (11). Understanding the relationship between CRP levels and mortality in arthritis patients could inform health education and promotion strategies aimed at reducing inflammation and improving long-term outcomes.

The China Health and Retirement Longitudinal Study (CHARLS) provides a unique opportunity to explore this relationship using nationally representative data on the health, socioeconomic status, and mortality of the Chinese older adult population. By leveraging the CHARLS database, this study aims to examine the association between CRP levels and all-cause mortality in arthritis patients. The findings of this study could contribute to the development of public health education and promotion programs that emphasize the importance of inflammation management, health literacy, and early intervention strategies to improve survival outcomes and reduce the public health burden of arthritis.

## 2 Materials and methods

### 2.1 Study design

The China Health and Retirement Longitudinal Study (CHARLS) is a nationally representative cohort study conducted by the National Development Research Institute of Peking University. Designed to minimize sampling bias and enhance representativeness, CHARLS employs a rigorous multi-stage sampling methodology. The study focuses on individuals aged 45 and older residing in mainland China, aiming to create a high-quality public database that integrates socio-economic, health, and mortality data. Initiated in 2011, the baseline survey is followed by biennial or triennial follow-up assessments. The study spans 150 county-level units and 450 village-level units, encompassing approximately 17,000 individuals from 10,000 households. This comprehensive dataset provides a valuable resource for exploring public health challenges and informing health education and promotion strategies.

### 2.2 Participants

**Figure 1:**
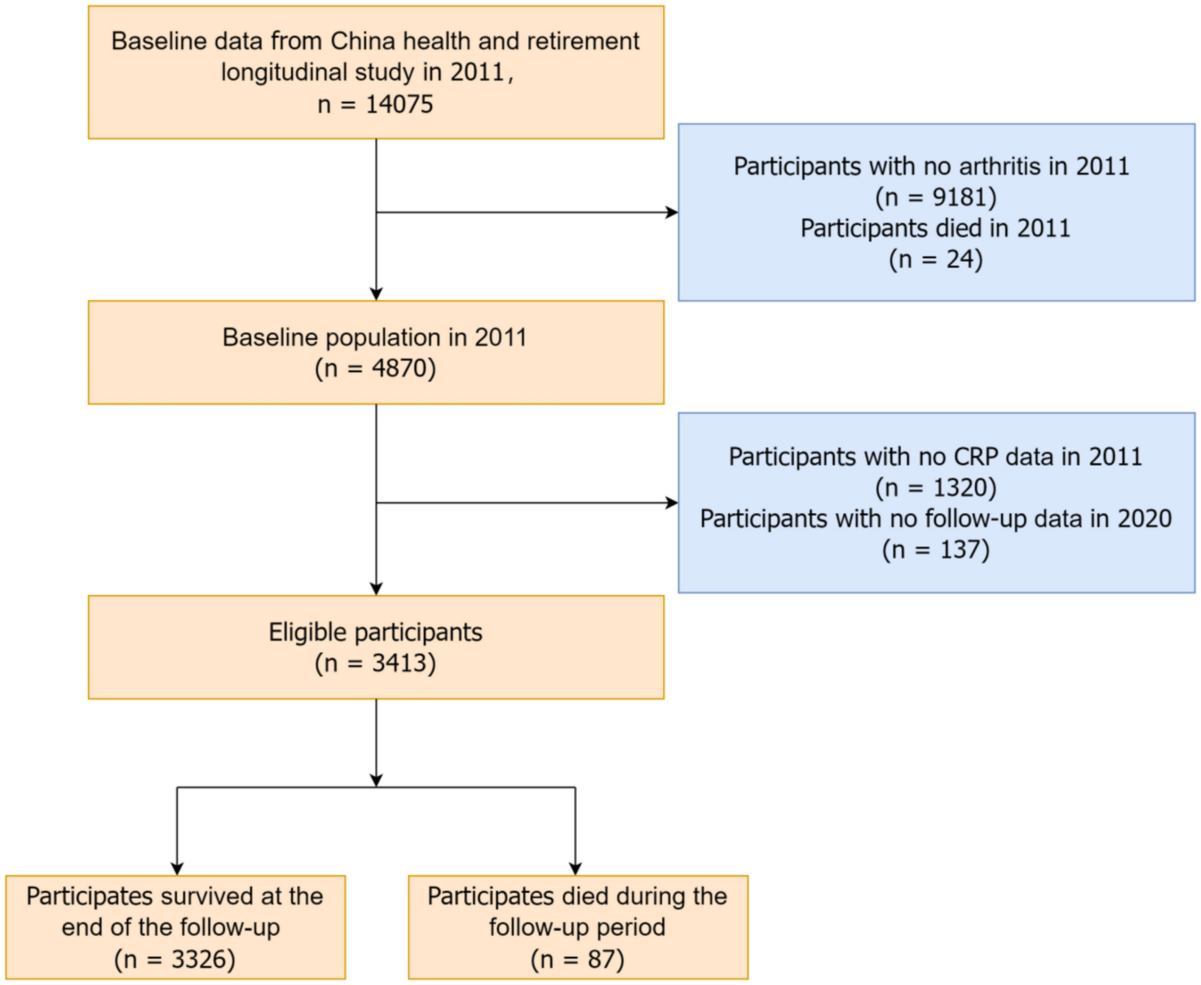
Flow chart of participant screening.

This study utilized data from the 2011 CHARLS baseline survey, focusing on arthritis and mortality-related variables. The initial sample included 14,075 participants. We excluded 9,181 participants without arthritis and 24 participants who died in 2011. Follow-up interviews were conducted in 2013, 2015, 2018, and 2020. After excluding 1,320 participants without CRP data and 137 participants without follow-up data, the final analysis included 3,413 participants. Arthritis was defined based on self-reported responses to the CHARLS questionnaire, which asked participants, “Have you been diagnosed with arthritis?” A “yes” response classified participants as having arthritis, encompassing both osteoarthritis (OA) and rheumatoid arthritis (RA). Figure 1 illustrates the detailed recruitment flowchart of the study subjects.

### 2.3 Variables

#### 2.3.1 Primary outcome

The primary outcome variable was all-cause mortality among arthritis patients, assessed during follow-up interviews in 2013, 2015, 2018, and 2020. Exact death dates were available for 2013 and 2020, while survival time for other cases was estimated using the median time from the baseline interview to the year of death.

#### 2.3.2 Primary exposure

The primary exposure variable was C-reactive protein (CRP) levels, categorized into four groups based on concentration:

Less than 1 mg/L.

Greater than or equal to 1 mg/L and less than 2 mg/L.

Greater than or equal to 2 mg/L and less than 3 mg/L.

Greater than or equal to 3 mg/L.

#### 2.3.3 Covariates

Other laboratory indicators included white blood cell count (WBC), hemoglobin (HGB), platelet count (PLT), glycosylated hemoglobin (HbA1c), creatinine (Cr), cystatin C, uric acid (UA), total cholesterol (TC), high-density lipoprotein (HDL), low-density lipoprotein (LDL), and triglycerides (TG). Estimated glomerular filtration rate (eGFR) was calculated based on creatinine and cystatin C, excluding race (12).

Demographic and socioeconomic characteristics included age, gender, ethnicity, marital status, education level, and residence (rural or urban). Lifestyle factors included smoking (defined as smoking over 100 cigarettes in a lifetime), alcohol consumption, body mass index (BMI), and metabolic equivalent (MET). Comorbidities such as hypertension, diabetes (DM), cancer, and cardiovascular disease (CVD) were also considered. Chronic diseases were assessed based on self-reported diagnoses by a doctor, with responses limited to “yes” or “no.”

### 2.4 Statistical analysis

All statistical analyses were conducted using R version 4.4.1 (http://www.r-project.org), with statistical significance set at *p* < 0.05. Baseline characteristics were summarized as mean (standard error) for continuous variables and sample size (percentage) for categorical variables.

Kaplan–Meier survival curves were used to illustrate mortality rates across different CRP levels, while Cox proportional hazards regression models were employed to explore the association between CRP levels and all-cause mortality. Hazard ratios (HRs) and 95% confidence intervals (CIs) were reported.

To assess the impact of covariates, three Cox models were constructed:

Model 1: Unadjusted.

Model 2: Adjusted for demographic and socioeconomic characteristics with *p*-values <0.10, including age, sex, marital status, and educational level.

Model 3: Further adjusted for additional factors with p-values <0.10, including age, sex, marital status, educational level, smoking, hypertension, CVD, cancer, diabetes, BMI, MET, eGFR, WBC, HGB, and UA. Diabetes was included in Model 3 due to its established role as a high-risk factor for mortality in arthritis patients. Since eGFR was already included as a kidney function indicator, creatinine and cystatin C were excluded from Model 3 to avoid multicollinearity.

## 3 Results

A total of 3,413 participants were included in the study, with 87 deaths recorded over the 10-year follow-up period. Compared to survivors, the deceased group exhibited distinct demographic, socioeconomic, and health-related characteristics. Specifically, the deceased group had higher proportions of males, never-married individuals, and those with lower education levels (elementary school and below). They were also more likely to be smokers and to have comorbidities such as diabetes, cancer, and cardiovascular disease (CVD). Additionally, the deceased group was older and had elevated levels of C-reactive protein (CRP), creatinine (Cr), and uric acid (UA), but lower body mass index (BMI), metabolic equivalent (MET), estimated glomerular filtration rate (eGFR), and hemoglobin (HGB). No significant differences were observed between the groups in terms of ethnicity, residence (rural or urban), alcohol consumption, white blood cell count (WBC), platelet count (PLT), glucose (GLU), glycosylated hemoglobin (HbA1c), total cholesterol (TC), triglycerides (TG), high-density lipoprotein (HDL), and low-density lipoprotein (LDL) (*p* > 0.05). Table 1 provides a detailed summary of the baseline characteristics.

**Table 1:** Characteristics of participants.

Variable	Total (*n* = 3,413)	Survive (*n* = 3,326)	Death (*n* = 87)	*p* value
**Age**	58.64 ± 8.71	58.38 ± 8.55	68.76 ± 9.03	**<0.0001**
**Sex**				**<0.01**
Male	1,319(38.68)	1,271(38.25)	48(55.17)	
Female	2091(61.32)	2052(61.75)	39(44.83)	
**Race**				0.89
Han	3,025(90.98)	2,997(90.96)	28(93.33)	
Others	300(9.02)	298(9.04)	2(6.67)	
**Marital status**				**<0.0001**
Non-married	399(11.69)	374(11.24)	25(28.74)	
Married	3,014(88.31)	2,952(88.76)	62(71.26)	
**Educational level**				**0.04**
Elementary school and below	2,596(76.13)	2,520(75.84)	76(87.36)	
High school	789(23.14)	778(23.41)	11(12.64)	
College and higher	25(0.73)	25(0.75)	0(0.00)	
**Residence place**				0.99
Rural	2,375(69.59)	2,315(69.60)	60(68.97)	
Urban	1,038(30.41)	1,011(30.40)	27(31.03)	
**Smoke**				**<0.01**
No	2,275(66.66)	2,231(67.08)	44(50.57)	
Yes	1,138(33.34)	1,095(32.92)	43(49.43)	
**Alcohol**				0.72
No	2,392(70.11)	2,329(70.05)	63(72.41)	
Yes	1,020(29.89)	996(29.95)	24(27.59)	
**Hypertension**				**<0.001**
No	2,470(73.08)	2,421(73.52)	49(56.32)	
Yes	910(26.92)	872(26.48)	38(43.68)	
**Diabetes mellitus (DM)**				0.17
No	3,185(94.62)	3,106(94.72)	79(90.80)	
Yes	181(5.38)	173(5.28)	8(9.20)	
**Cancer**				**<0.01**
No	3,358(99.09)	3,275(99.18)	83(95.40)	
Yes	31(0.91)	27(0.82)	4(4.60)	
**Cardiovascular disease (CVD)**				**<0.01**
No	2,830(83.09)	2,768(83.40)	62(71.26)	
Yes	576(16.91)	551(16.60)	25(28.74)	
**Body mass index**	23.72 ± 3.95	23.76 ± 3.95	22.09 ± 3.72	**<0.001**
**Metabolic equivalent (MET)**	9267.37 ± 7357.25	9344.17 ± 7367.87	5245.00 ± 5544.03	**<0.01**
**C-reactive protein (CRP)**	2.65 ± 6.59	2.55 ± 6.33	6.52 ± 12.45	**<0.01**
**WBC**	6.24 ± 1.86	6.23 ± 1.86	6.63 ± 2.05	0.07
**HGB**	14.25 ± 2.19	14.26 ± 2.19	13.69 ± 2.02	**0.01**
**PLT**	213.05 ± 78.41	212.92 ± 77.71	217.93 ± 102.14	0.65
**HbA1c**	5.27 ± 0.75	5.27 ± 0.75	5.26 ± 0.76	0.87
**Estimated glomerular filtration rate (eGFR)**	96.22 ± 13.99	96.49 ± 13.75	85.72 ± 18.63	**<0.0001**
**Creatinine (Cr)**	0.76 ± 0.21	0.76 ± 0.19	0.90 ± 0.58	**0.03**
**CystatinC**	1.00 ± 0.26	0.99 ± 0.25	1.24 ± 0.48	**<0.0001**
**Uric acid (UA)**	4.36 ± 1.20	4.35 ± 1.19	4.75 ± 1.46	**0.01**
**Total cholesterol (TC)**	195.37 ± 39.32	195.43 ± 38.94	193.06 ± 52.01	0.67
**HDL**	51.65 ± 15.25	51.64 ± 15.22	52.25 ± 16.40	0.73
**LDL**	116.83 ± 34.81	116.99 ± 34.74	110.92 ± 37.27	0.14
**Triglyceride (TG)**	138.46 ± 125.71	138.12 ± 123.41	151.17 ± 194.79	0.54

Bold values indicate a *p*-value less than 0.05, which means these results are statistically significant.

### 3.1 Association between CRP levels and mortality risk

Cox proportional hazards regression analysis demonstrated a significant association between CRP levels and all-cause mortality risk among arthritis patients across all three models. In Models 1, 2, and 3, a linear relationship was observed between CRP levels and mortality risk (*p* < 0.0001, p < 0.0001, and *p* = 0.04, respectively). When CRP was treated as a categorical variable (<1, 1–2, 2–3, ≥3 mg/L), patients with CRP levels ≥3 mg/L exhibited a significantly higher risk of mortality compared to those with CRP levels <1 mg/L. The hazard ratios (HRs) for CRP ≥3 mg/L were as follows:

Model 1: HR = 3.73 (95% CI: 2.23–6.23).

Model 2: HR = 3.00 (95% CI: 1.79–5.01).

Model 3: HR = 4.94 (95% CI: 1.77–13.78).

Table 2 presents the detailed relationship between CRP levels and all-cause mortality among arthritis patients. The log-likelihood of the comprehensive model (Model 3), which included all significant confounding variables, was −100.8298.

**Table 2:** Association of CRP with arthritis.

Exposure	Model 1		Model 2		Model 3	
	95%CI	*p* value	95%CI	*P* value	95%CI	*P* value
**C-reactive protein (CRP)**	**1.03(1.02, 1.05)**	**<0.0001**	**1.03(1.02, 1.05)**	**<0.0001**	**1.04(1.01, 1.08)**	**0.01**
C-reactive protein (CRP)						
<1	Reference		Reference		Reference	
<2	1.59 (0.89, 2.85)	0.12	1.44 (0.80, 2.59)	0.22	0.59 (0.12, 2.95)	0.52
<3	1.57 (0.71, 3.47)	0.26	1.34 (0.60, 2.95)	0.47	0 (0.00, 14, 301, 243, 026, 944, 604)	0.76
> = 3	**3.73 (2.23, 6.23)**	**<0.0001**	**3.00 (1.79, 5.01)**	**<0.0001**	**4.94 (1.77, 13.78)**	**0.002**
*P* for trend		**0.03**		0.08		0.23

Model 1 was adjusted for no covariates.

Model 2 was adjusted for age, sex, marital status, educational level.

Model 3 was adjusted for covariates in model 2 plus smoke, hypertension, CVD, cancer, DM, BMI, MET, eGFR, WBC, HGB, UA. Bold values indicate a *p*-value less than 0.05, which means these results are statistically significant.

### 3.2 Kaplan–Meier survival analysis

**Figure 2:**
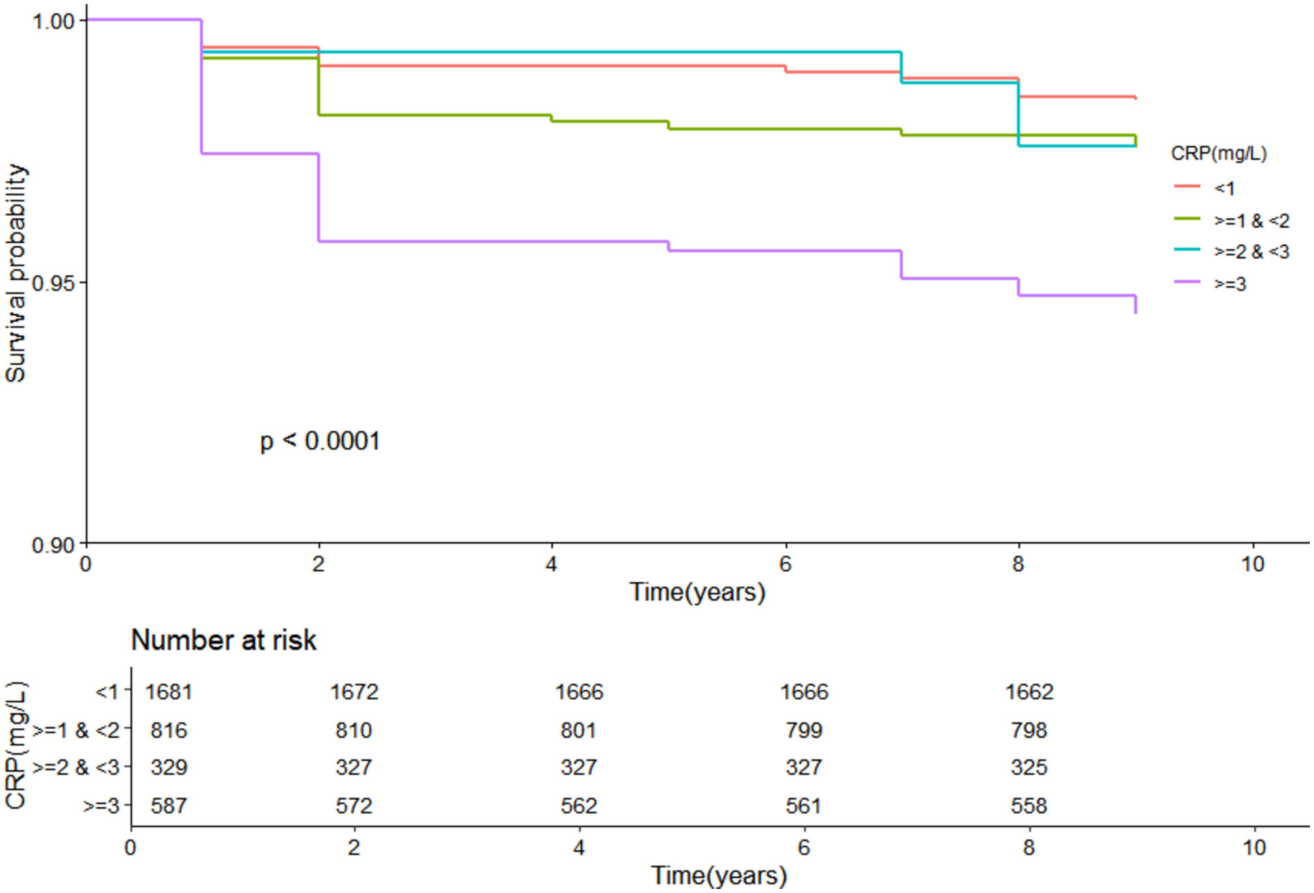
KM curve of CRP and all-cause mortality in arthritis patients.

Kaplan–Meier survival curves further corroborated the findings from the Cox regression analysis. Arthritis patients with CRP levels ≥3 mg/L demonstrated a markedly higher risk of mortality over the 10-year follow-up period compared to those with lower CRP levels. Figure 2 illustrates the survival curves, highlighting the significant disparity in survival outcomes based on CRP levels.

## 4 Discussion

This study, based on a large nationwide cohort involving 17,000 participants from most provinces in China, demonstrates that higher serum CRP levels in arthritis patients are significantly associated with increased all-cause mortality, even after adjusting for potential confounders. These findings highlight the importance of addressing systemic inflammation in arthritis patients, not only as a clinical concern but also as a public health priority. By identifying CRP as a practical biomarker for mortality risk, this study provides valuable insights for public health education and promotion strategies aimed at improving health outcomes in this vulnerable population.

CRP, an acute-phase protein, is a well-established marker of systemic inflammation and is associated with various chronic diseases, including cardiovascular disease (CVD) and metabolic disorders (13–15). Previous studies have demonstrated a strong correlation between elevated CRP levels and mortality risk (16–18). For example, elevated high-sensitivity CRP concentrations have been linked to increased all-cause mortality among the older adult in China (19). Similarly, a U.S. study found that men with CRP >3.0 mg/L faced higher cardiovascular and all-cause mortality risks compared to those with CRP ≤3.0 mg/L, although this association was less pronounced in women (20). In arthritis populations, elevated CRP has been identified as a predictor of increased mortality risk, including in osteoarthritis (21) and gout patients (22). Furthermore, Zhai et al. reported that all-cause mortality in rheumatoid arthritis (RA) patients is positively associated with the inflammatory burden index (IBI), calculated as CRP × neutrophils / lymphocytes, using data from the NHANES database (23). Our findings align with these studies, reinforcing the role of CRP as a critical predictor of mortality risk in arthritis patients.

### 4.1 Public health implications

The association between elevated CRP levels and increased all-cause mortality in arthritis patients has significant implications for public health education and promotion. First, these findings underscore the need for targeted health education programs to raise awareness about the role of systemic inflammation in arthritis and its broader health consequences. Educating patients and healthcare providers about the importance of monitoring CRP levels and managing inflammation could lead to earlier interventions and improved health outcomes.

Second, the results highlight the potential for integrating CRP monitoring into community-based health promotion programs. For example, routine CRP screenings could be incorporated into workplace or community health initiatives, particularly for populations at higher risk of arthritis or chronic inflammation. Such programs could emphasize lifestyle modifications, including physical activity, balanced nutrition, and smoking cessation, which are known to reduce systemic inflammation and improve overall health.

Lastly, this study supports the development of evidence-based policies aimed at reducing the burden of chronic inflammation in arthritis patients. Policymakers could prioritize funding for public health campaigns that promote inflammation management and advocate for accessible healthcare services, including routine biomarker testing and personalized interventions. These efforts align with the Sustainable Development Goals (SDGs), particularly SDG 3 (Good Health and Well-being) and SDG 4 (Quality Education), by promoting health literacy and equitable access to care.

### 4.2 Potential mechanisms

The observed association between elevated CRP levels and increased all-cause mortality in arthritis patients may be explained by several mechanisms. First, higher CRP levels may exacerbate oxidative stress, leading to cellular and tissue damage, which accelerates aging and the progression of chronic diseases. Second, chronic inflammation, as indicated by elevated CRP levels, can have systemic effects beyond joint health, including adverse impacts on cardiovascular and metabolic systems, thereby increasing mortality risk. Third, elevated CRP levels may reflect higher disease activity in arthritis patients, which could worsen joint damage and contribute to the development or progression of comorbid conditions such as CVD and diabetes, ultimately affecting survival outcomes.

### 4.3 Limitations

Despite its strengths, this study has several limitations. First, the CHARLS database relies on self-reported data, which may introduce recall bias or inaccuracies. Second, although we adjusted for multiple potential confounders, unmeasured variables may still influence the observed associations. Third, the study population is specific to China, which may limit the generalizability of the findings to other populations. Future research should aim to validate these findings in diverse populations and explore the causal pathways linking CRP levels to mortality risk in arthritis patients. Randomized controlled trials or longitudinal cohort studies could provide more robust evidence to inform public health interventions.

## 5 Conclusion

This study demonstrates a significant association between elevated CRP levels and increased all-cause mortality in arthritis patients, emphasizing the importance of systemic inflammation as a public health concern. CRP levels, particularly those ≥3 mg/L, may serve as a practical biomarker for identifying high-risk individuals and guiding targeted interventions. From a public health perspective, controlling inflammation through health education, lifestyle modifications, and accessible healthcare services is a critical strategy for improving survival outcomes and quality of life in arthritis patients. Future research should focus on elucidating the biological mechanisms underlying this association and developing innovative, evidence-based interventions to reduce the burden of inflammation-related mortality in arthritis populations.

## Data Availability

The raw data supporting the conclusions of this article will be made available by the authors, without undue reservation.
